# Exploring potential EQ-5D bolt-on dimensions with a qualitative approach: an interview study in Hong Kong SAR, China

**DOI:** 10.1186/s12955-024-02259-6

**Published:** 2024-05-31

**Authors:** Clement Cheuk Wai Ng, Annie Wai Ling Cheung, Eliza Lai Yi Wong

**Affiliations:** 1 The Jockey Club School of Public Health and Primary Care, Faculty of Medicine, The Chinese University of Hong Kong, Hong Kong SAR, China; 2 Centre for Health Systems and Policy Research, The Jockey Club School of Public Health and Primary Care, Faculty of Medicine, The Chinese University of Hong Kong, Hong Kong SAR, China; 3 Rm418, School of Public Health Building, Prince of Wales Hospital, Sha Tin, New Territories, Hong Kong SAR, China

**Keywords:** Health-related quality of life, EQ-5D, Bolt-on, Qualitative research

## Abstract

**Purpose:**

The introduction of bolt-on dimensions in EQ-5D instruments is growing common, but most bolt-on studies have targeted the diseased population and obtained bolt-on from other existing Health-related Quality of Life (HRQoL) instruments. As the qualitative approach offers important evidence to support the consistency and design of the potential bolt-on items, this paper studies the Hong Kong SAR community’s perception of the current EQ-5D-5 L instrument and identifies potential bolt-on via a qualitative approach.

**Methods:**

A representative sample mix was recruited based on the age group, gender, and education level composition of the Hong Kong SAR community by quota sampling. Semi-structured interviews were conducted and the interviews were transcribed and coded to identify emergent and recurrent themes.

**Results:**

Thirty interviews were conducted and the majority of the interviewees considered the EQ-5D-5 L insufficiently comprehensive to illustrate their HRQoL. While some key HRQoL aspects included in the EQ-5D matched with the community’s HRQoL perception, respondents showed concern about the potential overlap of the existing HRQoL dimension, the optimal number or attributes, and the appropriateness of the EQ-VAS. Among the potential bolt-on dimensions that emerged, ‘Sleep’, ‘Interpersonal Relationship’, and ‘Satisfaction’ were the key potential bolt-on dimensions identified and emphasized in the interviews.

**Conclusions:**

The qualitative findings of the study illustrate the possible gap between EQ-5D-5 L measurements and community HRQoL perception, while the findings support the development of EQ-5D bolt-on dimensions in the target community with content and face validity.

**Supplementary Information:**

The online version contains supplementary material available at 10.1186/s12955-024-02259-6.

## Background

With the rising global predominance of chronic disease conditions, a surging number of people dwell in communities with impaired physical, psychological, and social functioning but prolonged life expectancy [1]. As the concept of health-related quality of life (HRQoL) enables us to assess the patient-perceived health impact of medical conditions [2], HRQoL has become a popular patient-reported outcome (PRO) in recent years to supplement objective indicators to improve health outcomes [3]. In addition, the application of HRQoL may facilitate economic evaluations and decision-making at both the clinical and policy levels [4]. 

One of the most widely used generic HRQoL instruments is the EQ-5D. With the generic and brevity nature of the EQ-5D, the EQ-5D instrument can be administered at a low cost, while the utility derived can facilitate economic evaluations. However, the EQ-5D-5L is often prone to a high ceiling effect [5], and some of the significant HRQoL aspects might not be captured with the current dimensions [6]. Recent research considers the inclusion of bolt-on dimensions as a ‘promising solution’ to address such limitations [7]. An increasing number of publications explored the appendment of the EQ-5D bolt-ons which aim to enrich the health state classification, and to enhance the responsiveness and content validity of the EQ-5D [8–10]. 

As described by the EQ-5D bolt-on development guidelines, qualitative evidence of bolt-on development can support the design of clear and concise HRQoL descriptors [8]. However, a systematic review reported that many EQ-5D bolt-on studies obtained their bolt-on items directly from other existing generic HRQoL instruments such as the WHOQOL-BREF, Assessment of Quality of Life, and Health Utility Index, but only one project reported the identification and development of the EQ-5D bolt-on dimension via qualitative research [9]. While two other recent studies had attempted to develop EQ-5D disease-specific bolt-on qualitatively [11, 12], the majority of HRQoL instrument development research still adopted a quantitative approach, despite the qualitative scope providing another important perspective in HRQoL research [13]. 

In addition to the development strategy, most bolt-on studies focused on disease-specific populations [9], and scarce research had investigated the HRQoL construct perceived by the general population, especially in non-Western cultures [14, 15]. However, it is equally important to understand the possible discrepancy between HRQoL measurements and the community’s HRQoL perception [16], when healthcare services and systems are designed for both the ill and healthy people to sustain health.

The community-specific bolt-on item may vary according to community characteristics. The general public in the United Kingdom regarded sensory deprivation and mental health items as significant dimensions not covered by the EQ-5D [17], while respondents from New Zealand raised examples such as fitness, happiness, mental health, and cognition as areas not illustrated by the EQ-5D [18]. In particular, the current EQ-5D instrument may not illustrate the HRQoL perception in the Asian communities well with its European development background [16], and some Asian communities such as China, Korean, Malaysia and Thailand have been attempting to develop its cultural-specific bolt-ons [19–23]. In Hong Kong context, ceiling effect could be observed as 46% of the respondents reported [11,111] in the EQ-5D-5 L [24]. A local telephone survey further revealed that the current EQ-5D-5L may not be sensitive to reflect the change in patient-reported HRQoL, while the majority welcomed the inclusion of appetite, hearing, vision and energy/ sleeping quality to support better HRQoL illustration [25]. To understand and interpret the community’s HRQoL perception and screen for appropriate bolt-on dimensions, this study aimed to identify relevant EQ-5D bolt-on candidates with a qualitative approach in Hong Kong SAR, China.

## Methods

### Study design and participants

A qualitative individual interview study was conducted. Details of the study design, analysis, and findings were checked with the Consolidated Criteria for Reporting Qualitative (COREQ) 32-item checklist [26]. 

To ensure the heterogeneity of the sample population, quota sampling was applied with reference to local census data on age group, gender, and highest education level attained. Hong Kong Cantonese-speaking residents aged ≥ 18 were invited for the interview.

Sample recruitment was promoted openly in the community center, hospital, and campus areas, while some cases were approached by referral to fill in the designated quota. The number of total interviews conducted was determined by data saturation when no additional important themes were revealed from the new interviews.

### Data collection process

The interview focused on the EQ-5D-5L as the EQ-5D-5L offered improved sensitivity and discriminatory power [5, 27–29], and had been used for local healthcare service benchmarking [30, 31]. Before the interview, the participants completed the EQ-5D-5L(HK) survey after they provided written consent, allowing the participants to construct a general picture of the interview topic.

The interview was comprised three sections: (i) perception on ‘health’ and ‘HRQoL’; (ii) perception and comments on current EQ-5D-5 L(HK) instrument; and (iii) exploration of potential bolt-on(s) for EQ-5D-5L(HK). The first two interviews were regarded as pilot test to determine the appropriateness of the interview guide and data collection process. Participants were invited to explain their perceptions of ‘health’ and ‘HRQoL’ in the first section. In the second section, the interviewees were asked to comment on the validity of the current EQ-5D-5L(HK), and they were also encouraged to freely suggest any potential bolt-on that may enrich the current EQ-5D-5L. In the final section, the interviews were then invited to identify any other potential EQ-5D-5L bolt-on dimensions from the two lists of the candidates based on the past literatures. To minimize confirmation bias, the two lists were put at the end of the section and presented to the interviewees after they shared their personal views and suggestions on potential EQ-5D-5L bolt-on on the current EQ-5D-5L. The first 8-item list were prepared based on a factor analysis study of EQ-5D bolt-on dimensions and the local telephone survey [25, 32], while dimensions not covered by the EQ-5D and the first list were extracted from local validated WHOQOL-BREF(HK) as the second 12-item list [33, 34]. With the bolt-on candidates proposed, the interviewees were first invited to express their interpretation of the bolt-on, and then explain its potential improvement if the dimension is appended to the EQ-5D-5L(HK). Trained interviewers would queue the respondent to elaborate on what EQ-5D-5L(HK) gap or limitation may be addressed by the bolt-on candidates, and how would the bolt-on fit the community’s HRQoL perception. Ranking exercises were conducted, which interviewees explained and ranked the bolt-on dimensions from the most useful to least useful in their respective lists. Participants’ sociodemographic characteristics were collected at the end of interview. Each interview lasted approximately 45–60 min, and the interviews were audio-recorded under the participants’ consent.

### Data analysis

Audiotapes of the interviews were transcribed verbatim, and the data analysis was handled with Dedoose [35]. Applying thematic analysis referencing the interview guide, transcripts were coded twice by independent reviewers to identify emergent and recurrent themes. The reviewers cross-checked the codebooks before restructuring the codes into master themes or subthemes. Disagreements between codes were resolved by discussion among the research team, and interview excerpts were quoted to illustrate the respective themes. The results of the ranking exercise were summarised by (i) the frequency of the dimension chosen as top three in its list; (ii) summation of the ranked votes, and (iii) the relative ranking reflected by ii, with lower total ranked indicating higher priorities by the respondents.

## Results

### Sample characteristics

Thirty face-to-face interviews were conducted between March and August 2021. The sample mix was similar to the Hong Kong demographic composition in terms of age group, gender, and highest education attained [36]. Similar to local statistics, approximately one-third of the sample suffered from chronic conditions [37]. Sample characteristics are summarized in Table 1.

Considering the EQ-5D-5L, 40% of respondents experienced a certain extent of Anxiety/Depression (AD) problem, while most experienced no problems in other EQ-5D dimensions. The mean EQ-5D-5 L utility and EQ-VAS were 0.902 and 78.5 respectively, which were slightly lower than the population norm in 2015 [38]. EQ-5D-5L responses of the samples area tabulated in Table 2.

**Table 1:** Characteristics of the sample population

	Study Sample *n* (%)	HK Population (%)^a^
**Age Group**		
18–24^#^	5 (16.7)	(12.6)
25–44	8 (26.7)	(31.9)
45–64	11 (36.7)	(36.8)
≥65	6 (20.0)	(18.8)
**Gender**		
Female	16 (53.3)	(55.0)
Male	14 (46.7)	(45.0)
**Marital Status**		
Single	12 (40.0)	-
Married/ Co-living	13 (43.3)	-
Divorced/ Separate	2 (6.7)	-
Widowed	3 (10.0)	-
**Highest Education Attainment**		
Primary School or Below	8 (26.7)	(26.4)
Secondary/ Sub- degree	12 (40.0)	(42.5)
Degree or above	10 (33.3)	(31.1)
**Job Status**		
Full-time	21 (70.0)	-
Part-time	5 (16.7)	-
Retired /Unemployed	4 (13.3)	-
**Self-reported Chronic Disease(s)**		
Yes	11 (36.7)	-
No/ Uncertain	19 (63.3)	-
**Total**	30(100)	

a: Hong Kong Population By-census Data

#: Data for Hong Kong Census referred to the population aged ≥ 15 years but the study sample focused on aged ≥ 18 years

**Table 2:** EQ-5D-5L profiling of the sample population

EQ-5D Dimension	Study Sample *n* (%)
**Mobility**	
Level 1	28 (93.3)
Level 2	1 (3.3)
Level 3	1 (3.3)
Level 4	0 (0)
Level 5	0 (0)
**Self-care**	
Level 1	29 (96.7)
Level 2	1 (3.3)
Level 3	0 (0)
Level 4	0 (0)
Level 5	0 (0)
**Usual Activities**	
Level 1	27 (90.0)
Level 2	2 (6.7)
Level 3	1 (3.3
Level 4	0 (0)
Level 5	0 (0)
**Pain/ Discomfort**	
Level 1	27 (90.0)
Level 2	2 (6.7)
Level 3	1 (3.3)
Level 4	0 (0)
Level 5	0 (0)
**Anxiety/ Depression**	
Level 1	18 (60.0)
Level 2	10 (33.3)
Level 3	2 (6.7)
Level 4	0 (0)
Level 5	0 (0)
**EQ-5D-5 L Utility**	0.902
**EQ- VAS**	Mean: 78.5

### Perceptions of ‘Health’ and ‘HRQoL’

Interviewee discussed ‘health’ as a combination of physical, mental and social well-being, while often emphasized physical and mental health in their elaborations.

*“… I will look into physical and mental health first. Social well-being is… a factor affecting the two (physical and mental health), that’s what I think.”* H014.

*“In general, …divided into physical and mental aspects. In physical (health), there is no disease or pain, and you have a routine daily lifestyle… and pattern. On the mental (health) side, you should feel motivated and anxious… not exactly anxiety-free but shouldn’t pressure you too much.”* H023.

For HRQoL, respondents repeatedly reported ‘pain-free and disease-free’(*n* = 18), ‘eat well’(*n* = 13), ‘walk well’(*n* = 12), ‘move well’(*n* = 10), ‘sleep well’(n = 8) as the common HRQoL targets that the community focused on, which some were highly similar to the EQ-5D-5L dimensions such as Mobility (MO) and Usual Activities (UA). Interviewees further emphasized how unhealthy habits shared by the Hong Kong community such as “irregular sleep schedule” and “working overtime” may deteriorate HRQoL.

*“(If you) walk well, move well, eat well, sleep well, and everything (HRQoL) would be fine, you may even include exercise too.”* H007.

*“You don’t truly have to consider social well-being, or anxiety and mental health-related factors. Naturally your mood, your own self, physical (health), you would be relaxed or feel better if you eat well, sleep well, walk well and move well”* H030.

### The validity of the current EQ-5D-5L

Most participants considered the 5-level design appropriate for illustrating changes in HRQoL measurements and health state classifications. However, the majority of participants (66.7%, *n* = 20) stated that the five dimensions were not comprehensive enough to describe their HRQoL.

Many interviewees were comfortable with the current length of the EQ-5D-5L, but also considered 5–10 questions as adequate, while a couple of respondents suggested 20. However, 19 participants raised concerns about the overlapping between the original EQ-5D dimensions, and some struggled if such a design may cause confusion and redundancy.

*“I would prefer more (question items)… as I paid emphasis on mental health. Within the five (EQ-5D) dimensions, seems only anxiety/depression may address psychological health, and I think we need more items (for mental health).”* H018.

*“This instrument focuses on physical health, and lacks coverage in mental and social well- being.”* H023.

*“Usual activities and mobility … actually these three (self-care) are slightly duplicated… Your movements, maybe daily moving, standing and sitting…, in these three (EQ-5D dimensions) maybe… I can’t tell if there is more than enough information or a duplication.”* H030.

Several participants considered the EQ-VAS as a wrap-up which they tended to respond by ‘gut feeling’ and out of intuition. Some participants preferred reserving rooms on both ends for unforeseeable circumstances such as accidents and undiagnosed conditions. Respondents considered wide range of factors when answering the EQ-VAS, and many highlighted factors were not necessarily HRQoL-specific, examples include weather of the day, unforeseeable circumstances, or other sociodemographic factors.

*“I think the scoring (VAS) is not accurate and not very convincing… I could be very well in terms of biological indicators today… but with any incidents today… (I could get) very upset… such as the death of a family member… but with the scale alone… it would be hard to tell whether you experienced difficulty in biologically, or physiologically… it is just too general.”* H018.

*“Nice weather would make a difference (in VAS), like extra points will be added for finer weather… and the next would be having a smooth day at work, so you won’t be working late, or be too late when you have your rest.”* H022.

Exploration of the Potential EQ-5D-5 L Bolt-on Dimension.

Four interviewees proposed ‘exercise’ as a as possible supplement to the EQ-5D-5 L respectively, but had later probed ‘exercise’ into a proxy of MO and UA. Similarly, three sample suggested ‘job’ as a bolt-on candidate, but later considered ‘job’ already covered by a descriptor in UA. Otherwise, the bolt-on candidates discussed by the respondents could be represented by the dimensions included in the two EQ-5D-5L potential bolt-on lists. Many interviewees found the list based on the EQ-5D bolt-on factor analysis more relevant and compatible, and considered dimensions from the WHOQOL-BREF-HK to be vague and distant to the original EQ-5D.

*“(adding items from the WHOQOL-BREF HK) doesn’t have much impact… some are repeated… or subdividing extra details from the existing list. Some (items from the WHOQOL-BREF HK) are meaningless to the current instrument (EQ-5D-5L) too. As for myself… these items couldn’t apply to someone like me, it wouldn’t be useful asking me these (as bolt-ons)”* H028.

The results of the ranking exercise with potential bolt-on dimensions discussed in the EQ-5D list are reported in Table 3. The priority was topped by ‘Sleep’, followed by ‘Interpersonal Relationships’, ‘Energy’, ‘Satisfaction’, ‘Appetite’, ‘Speech/ Cognition’, ‘Vision’ and ‘Hearing’ in descending order.

Among all potential bolt-ons that emerged, almost all interviewees (*n* = 29) discussed ‘Sleep’ (SL) as an EQ-5D-5 L bolt-on candidate and SL recorded the highest coding frequency. Interviewees often drew a cyclic relationship between SL and ‘energy’, and proposed the combination of the two which was adherent with Finch [32]. Respondents intended to consider SL as bolt-on candidate to enrich the mental health description in the EQ-5D-5L(HK), as they considered the SL as an important HRQoL dimension to illustrate the daily recovery process and functional status not covered by the current EQ-5D-5L(HK).

*“I believe sleep is an important criterion for the Hong Kong people, because… everyone knows Hong Kong people are fast-paced, everything seems tense. In my opinion, when I glanced through the list and came across ‘sleep’, I would think yes, if we include sleep (into EQ-5D), given that…I can go to work, can take of myself, I don’t feel discomfort, or any depression, but actually… in fact, I heard a lot of friends not sleeping well including myself, so sleep is significant (to HRQoL).”* H011.

*“Sleeping Quality may reflect your mental health, on top of our answers… to (EQ-5D-5 L) anxiety/depression. These are one of the direct HRQoL indicators, even if your body… had no problem in your basic mobility, sleeping quality describes more than the physical but an overall condition. ”* H023.

‘Interpersonal Relationship’ (IR) was discussed by 24 interviewees as another potential EQ-5D bolt-on dimension to supplement the ‘social well-being’ gap perceived in the EQ-5D-5L. The interviewees highlighted that original five dimensions took heavy emphasis on the individual but paid little emphasis on how social engagements in daily life may enhance HRQoL. An IR bolt-on may take account of the gregarious nature of humans, and serve as an appropriate EQ-5D-5L bolt-on to reflect mental and social well-being.

*“the (EQ-5D-5L) five dimensions lack items on social or interpersonal relationships, social well-being… and it seems the most important (bolt-on). The appendments enriches (EQ-5D-5L), as it provides a whole new perspective.”* H013.

*“Interpersonal relationship discussed how man-to-man interact, these (EQ-5D-5L) questions are often individual-based, and if an individual has a normal social life, I would think… directly affect oneself, partially on the mental health dimensions…”* H017.

**Table 3:** Summary of the ranking exercise with the potential bolt-on items on the EQ-5D List

	Cumulative Top 3 Count	Total Ranked Votes	Relative Ranking By Total Ranked Voting
Sleep	22	78	1
Interpersonal Relationships	17	115	2
Energy	13	118	3
Satisfaction	14	129	4
Appetite	8	139	5
Speech/ Cognition	9	145	6
Vision	5	176	7
Hearing	7	180	8

After the considering SL and ‘energy’ together, ‘satisfaction’(SF) is the next prevalent bolt-on discussed by 19 interviewees to enhance the coverage on mental and social HRQoL. Respondents emphasized that the understanding of SF could differ widely between individuals, and these variations would impact immensely on the community’s HRQoL perception. Many interviewees preferred not to fix a coherent definition, and allowed SF as a malleable bolt-on candidate to cover intangible gaps uncaptured by the EQ-5D-5L instrument.

*“Satisfaction refers to how you perceive yourself as satisfied with different aspects such as your surroundings, family or money. Because I believe if someone is not feeling satisfied, he or she cannot not be considered as truly healthy both physically and mentally.”* H010.

*“Everyone’s definition of satisfaction differs, but if you look into the (EQ-5D-5L) five dimensions, four of them are very straightforward. For example, mobility, being able to self-care or not, or experiencing any pain. In fact, these (EQ-5D dimensions) are very direct, but for satisfaction, everyone may perhaps perceive it differently, and how it affects one’s emotion.”* H013.

*“This (EQ-5D-5L) tool has limited coverage on emotions, and the ‘Satisfaction’ dimension appears to me as enhancing the description of emotions and mental well-being, and would be a great enrichment to the (EQ-5D-5L) instrument at first impression.”* H017.

## Discussion

This study reported the Hong Kong SAR community’s perception of the EQ-5D-5L extensively, and although the EuroQol did not intend to cover all dimensions of health with the original EQ-5D descriptive structure [39, 40], the sample population welcomed the appendment of bolt-on dimensions to illustrate their HRQoL more comprehensively. The findings from the qualitative interview and the ranking exercise supported SL, IR, and SF as key potential EQ-5D-5L bolt-on dimensions for the Hong Kong SAR community. As the Hong Kong SAR-specific bolt-on candidates showed variance from other or neighboring communities, the study findings highlighted that the appropriate EQ-5D-5L bolt-on dimensions may vary according to community culture or characteristics, and the bolt-on development should be investigated and validated within the context of the target population. The qualitative approach with semi-structured interview allowed researchers to uncover suitable EQ-5D-5L bolt-on candidates with direct reference to the community’s perception of the HRQoL construct and provided evidence in future item design with the content and face validity gained from the target population [41–43]. Psychometric properties of the shortlisted EQ-5D-5 L bolt-on items should be examined quantitatively in future study, and valuation research may be conducted if appropriate bolt-on dimension(s) is validated. Despite the conventional EQ-VT valuation protocol may not be adopted for bolt-on valuation, a few publications had attempted to explore bolt-on valuation to facilitate economic evaluations [44–46]. 

The Hong Kong SAR community emphasized on both physical and mental health while some local HRQoL target could be covered by the original EQ-5D-5 L. However, other HRQoL targets such as *‘eat well and ‘sleep well’* may not be described distinctively by the current EQ-5D system. Careful interpretation should be made to determine whether these uncaptured HRQoL aspects should be designed as EQ-5D bolt-on dimensions, or supplemented as extra example descriptors to the existing dimensions in the local-adapted EQ-5D-5 L. As the EQ-5D-5L was designed as a generic instrument, amendments to its current form may not be preferred. Referencing the Thai example, ‘activities related to knee bending’ was regarded as a subtheme of UA, but was concurrently tested as a cultural-specific EQ-5D bolt-on [19, 20]. In contrast, this study had in-depth discussed the bolt-on candidates with direct reference to the EQ-5D-5L design, and the findings uncovered would go beyond ‘key HRQoL aspects’ but explicitly as ‘EQ-5D-5L bolt-on candidates’ as addressed in the second and third interview sections.

The study revealed that the sample population’s acceptance on increasing the number of relevant HRQoL attributes in the EQ-5D-5L. However, unassisted respondents could face difficulty in information processing and engagement if the instrument is too complex, and the ideal length for self-reported instruments was previously recommended as seven attributes [47]. Though support could be further provided if necessary [48], the final number of attributes in a HRQoL instrument should be carefully considered. Depending on the target population and desirable outcome, researchers should examine the advantages of using the EQ-5D and relevant bolt on(s), and choose the optimal tool of appropriate length and comprehensiveness for their research.

This study provided two lists of potential EQ-5D bolt-on items derived from an EQ-5D-themed factor analysis study and from the WHOQOL-BREF (HK) instrument for discussion [32, 34], and the respondents reported inclined preference to the former as they found the dimensions from the factor analysis more relevant and compatible to the EQ-5D-5L instrument. However, most previous studies directly adopted and tested bolt-on(s) obtained from existing HRQoL instruments [9], and findings from this study may hint that such a bolt-on development strategy may not be ideal. While such an approach could be a convenient way to test HRQoL areas not covered by the current EQ-5D, the choice of dimension and item design may need further verification before adoption in the target populations.

Currently, the statistical method for bolt-on validation has yet to be standardized, but many bolt-on publications had referenced the EQ-VAS responses to illustrate the improvements of introducing bolt-on dimensions [20, 23, 44, 49–53]. Yet, our study revealed that many respondents had considered non-HRQoL factors such as weather and unforeseeable circumstances in their EQ-VAS responses. As the EQ-VAS had been measuring a much boarder concept than the EQ-5D oftentimes [54, 55], EQ-VAS may not be a ‘golden standard’ in evaluating the impact of bolt-on appendment. This further implies that a standardized protocol for EQ-5D bolt-on development and validation is needed, while the performance of bolt-on could be reviewed by a range of criteria such as content validity, internal consistency, construct validity, ceiling effects, and responsiveness [8, 56]. 

The choice and performance of EQ-5D bolt-on candidates may vary across sample populations with different backgrounds. For example, sleeping quality was previously reported as an important HRQoL aspect in China [14], and sleep was considered a significant EQ-5D bolt-on candidate in Korea [23], where poor sleeping quality was shown associated with deteriorated EQ-5D utility [57]. In contrast, limited benefits of introducing a ‘sleep’ dimension in EQ-5D-3L were observed in England [50]. It would be crucial for bolt-on development to be validated in the target population, whereas further research on psychometric impacts and dimension structure should also be conducted on top of face validation before applying the bolt-on dimension in the target community [8]. Though most EQ-5D　bolt-on projects focused on disease-specific context, findings of this project may demonstrate the exploration of EQ-5D-5L bolt-on in community setting, while the discrepancy between EQ-5D-5L and community’s HRQoL perception observed may suggest further HRQoL instrument evaluation in other community setting to improve accuracy and comprehensiveness of HRQoL measurements.

This study has its limitations. First, despite obtaining face validity from the sample to combine SL and ‘energy’, the merging of two HRQoL aspects may conceal the possible interactions in measurements. Further research is necessary to study the effect of such merge. Besides, the dimensions obtained from the factor analysis studied were not translated by professional translators. Though the translated terminology was pilot-tested with a telephone survey [25], full translation may be preferred to develop comparable results with future EQ-5D bolt-on studies.

This study serves as an early attempt to develop community-specific EQ-5D-5L bolt-ons, and the qualitative approach provides in-depth evidence that SL, IF, and IR are considered potential EQ-5D-5L bolt-on dimensions in Hong Kong SAR. In contrast to the conventional ‘top-down’ approach of proposing bolt-on candidates by healthcare professionals [14], this study elicited ‘EQ-5D bolt-on candidates’ directly from the perspective of the general population, which may further enhance the validity of the EQ-5D-5L as a PRO instrument. The psychometric benefit of introducing these community-specific EQ-5D-5L bolt-ons should be investigated in future quantitative research.

### Electronic supplementary material

Below is the link to the electronic supplementary material.

Supplementary Material 1

## Data Availability

The datasets generated and/or analysed during the current study are available from the corresponding author on reasonable request.
