# Supplementary material for: Exploring potential EQ-5D bolt-on dimensions with a qualitative approach: an interview study in Hong Kong SAR, China
Source: Health Qual Life Outcomes. 2024 May 31;22:42. doi: 10.1186/s12955-024-02259-6 (PMC11141055; doi:10.1186/s12955-024-02259-6)
Supplement: Supplementary file 1 — Supplementary Material 1 [file 12955_2024_2259_MOESM1_ESM.docx]

**Appendix 1:** Consolidated criteria for reporting qualitative (COREQ) 32-item checklist

|  | Guide Question/ Description | Remarks | Line No. |
| --- | --- | --- | --- |
| **Domain 1: Research Team and Flexibility** | | | |
| Personal Characteristics | | | |
| 1. Interviewer/ Facilitator | What author/s conducted the interview/ focus group? | *“Data collection was handled by CCWN and AWLC.”* | 419-420 |
| 1. Credentials | What were the researcher’s credentials? | The credentials of the researchers were as follows.  Clement Cheuk Wai Ng: MPH (during data collection and analysis), PhD (at manuscript writing)  Annie Wai Ling Cheung: MPhil  Eliza Lai Yi Wong: PhD., MPH, RN | N.A. |
| 1. Occupation | What was their occupation at the time of the study? | The occupations at the time of the study were as follows.  Clement Cheuk Wai Ng: PhD Candidate  Annie Wai Ling Cheung: Research Associate  Eliza Lai Yi Wong: Professor | N.A. |
| 1. Gender | Was the researcher male or female? | The gender of the researchers was as follows.  Clement Cheuk Wai Ng: Male  Annie Wai Ling Cheung: Female  Eliza Lai Yi Wong: Female | N.A. |
| 1. Experience and Training | What experience or training did the researcher have? | The research team was experienced in Health-related Quality of Life (HRQoL) and Patient Experience researches in both qualitative and quantitative approaches. | N.A. |
| Relationship with Participants | | | |
| 1. Relationship Established | Was a relationship established prior to study commencement? | Only for research purpose, if the interviewee did not know any of the researcher personally prior to the data collection. | N.A. |
| 1. Participant Knowledge of the Interviewer | What did the participants know about the researcher? | All participants were aware that the interviewers were the staff from the Jockey Club School of Public Health and Primary Care. | N.A. |
| 1. Interviewer Characteristics | What characteristics were reported about the interviewer/ facilitator? | The research interest of the Principal Investigator was explained in the consent form, while the academic background of the interviewers was well-explained prior to the interview. | N.A. |

|  | Guide Question/ Description | Remarks | Line No. |
| --- | --- | --- | --- |
| Domain 2: Study Design | | | |
| Theoretical Framework | | | |
| 1. Methodological Orientation and Theory | What methodological orientation was stated to under pin the study? | Descriptive and thematic analysis were applied in the data analysis. | N.A. |
| Participant Selection | | | |
| 1. Sampling | How were participants selected? | *“To ensure the heterogeneity of the sample population, quota sampling was applied with reference to local census data on age group, gender, and highest education level attained.”* | 102-103 |
| 1. Method of Approach | How were participants approached? | *“Sample recruitment was promoted openly in the community center, hospital and campus area, while some cases were referred and approached purposively to fill in the designated quota.”* | 106-107 |
| 1. Sample Size | How many participants were in the study? | *“Thirty face-to-face interviews were conducted between March to August 2021.”* | 153 |
| 1. Non-participation | How many people refused to participate or dropped out? Reasons? | Adopting quota sampling, potential participants were recruited both openly and by referral simultaneously. A total of 31 participants had participated in the interview process and one was dropped out mid-way as she found it difficult to understand the topic under study. | N.A. |
| Setting | | | |
| 1. Setting of Data Collection | Where was the data collected? | The data collection was conducted in quiet areas in community centre, hospital or campus area of convenience and comfort to the participants. | N.A. |
| 1. Presence of Non-participants | Was anyone else present besides the participants and researchers? | During the data collection, only the participants and interviewers were present. | N.A. |
| 1. Description of Sample | What are the important characteristics of the sample? | *“To ensure heterogenicity of the sample population, quota sampling was applied with reference to local census data on age group, gender and highest education level attained.”*  *“Similar to local statistics, approximately one-third of the sample suffered from chronic conditions.”* | 102-103;  155-156 |

|  | Guide Question/ Description | Remarks | Line No. |
| --- | --- | --- | --- |
| Data Collection | | | |
| 1. Interview Guide | Were questions, prompts, guides provided by the authors? Was it pilot tested? | *“The interview was comprised three sections: i) perception on ‘health’ and ‘HRQoL’; ii) perception and comments on current EQ-5D-5L(HK) instrument; and iii) exploration of potential bolt-on(s) for EQ-5D-5L(HK). The first two interviews were regarded as pilot test to determine the appropriateness of the interview guide and data collection process..”* | 117-120 |
| 1. Repeat Interviews | Were repeat interviews carried out? If yes, how many? | No repeated interviews were conducted. | N/A |
| 1. Audio and Visual Recording | Did the research use audio or visual recording to collect the data? | *“…, and the interviews were audio-recorded under the participants’ consent.”* | 138 |
| 1. Field Notes | Were field notes made during and/or after the interview or focus group? | Field notes were made during the interview to enhance data interpretations. | N.A. |
| 1. Duration | What was the duration of the interviews or focus group? | *“Each interview lasted approximately 45-60 minutes…”* | 137-138 |
| 1. Data Saturation | Was data saturation discussed? | Data saturation was discussed to determine final sample size. | N.A. |
| 1. Transcripts Returned | Were transcripts returned to participants for comment | Transcripts were not returned to the participants for comments. | N.A. |

|  | Guide Question/ Description | Remarks | Line No. |
| --- | --- | --- | --- |
| Domain 3: Analysis and Findings | | | |
| Data Analysis | | | |
| 1. Number of Data Coders | How many data coders coded the data? | Three data coders were involved in this project. | N.A. |
| 1. Description of the Coding Tree? | Did authors provide a description of the coding tree? | The coding tree is broken down based on the three main interview sections as three main themes respectively, while each subtheme is addressed accordingly in the result section of the manuscript. | N.A. |
| 1. Derivation of Themes | Were themes identified in advance or derived from the data? | Based on the original EQ-5D design, recurrence of themes related to HRQoL dimensions such as ‘mobility’ and ‘pain/discomfort’ were anticipated and identified in advance. However, respondents were also encouraged to express any relevant ideas to reveal potential EQ-5D-5L bolt-ons, and the emergent items were derived as new themes uncovered from the data. | N.A. |
| 1. Software | What software, if applicable, was used to manage the data? | *“… and the data analysis was handled with Dedoose.”* | 141-142 |
| 1. Participant Checking | Did participants provide feedback on the findings? | Based on the study design, the interviewees did not provide feedback to the findings as the interview was one-off in nature. Alternatively, the interviewees would express their opinions on the potential bolt-on dimensions in the third section of the interview, followed by the ranking exercise. The interviewees were also invited to explain their rankings based on their interpretations of the bolt-on, and these feedbacks were audio-taped to support data analysis and confirm if the authors understand the samples’ responses. | N.A. |

|  | Guide Question/ Description | Remarks | Line No. |
| --- | --- | --- | --- |
| Reporting |  |  |  |
| 1. Quotations Presented | Were participants quotations presented to illustrate the themes/ findings? Was each quotation identified? | Quotations were provided to support the project findings and discussion of the manuscripts. Each quotation had credited the corresponding participant and their sample characteristics can be referenced in appendix. | N.A. |
| 1. Data and Findings Consistent | Was there consistency between the data presented and the findings? | The study findings were reported with the interview quotes to support the consistency. | N.A. |
| 1. Clarity of Major Themes | Were major themes clearly presented in the findings? | The interview comprised three sections and the discussion had addressed each of the components. | N.A. |
| 1. Clarity of Minor Themes | Is there a description of diverse cases or discussion of minor themes? | Illustrations of the minor or subthemes were supported by the interview excerpts quoted from different participants, while a representative sample mix was recruited by diverse age group, gender and highest education level attained. | N.A. |

**Appendix 2:** Summary of Interviewee Demographic Profile in Phase 2 Qualitative Interview

| ID | Gender | Age Group | Education | Marital  Status | Religion | Living Status | Ownership | Housing | Employment | Monthly Income | Chronic Disease |
| --- | --- | --- | --- | --- | --- | --- | --- | --- | --- | --- | --- |
| H001 | Female | 25-44 | Tertiary | Single | No | Living with Family | Private | Subsidised | Full-time | $30001-40000 | No |
| H002 | Female | 45-64 | Primary or Below | Married | No | Living with Family | Private | Subsidised | Full-time | $10001-20000 | Yes |
| H003 | Female | 25-44 | Tertiary | Co-habit | No | Living with Others | Private | Private | Full-time | $20001-30000 | No |
| H004 | Male | 45-64 | Tertiary | Married | No | Living with Family | Private | Private | Part-time | $30001-40000 | No |
| H005 | Male | 15-24 | Secondary | Single | Christian | Living with Family | Private | Private | Full-time | <$10000 | No |
| H006 | Male | 65+ | Secondary | Married | No | Living with Family | Rental | Public | Retired | <$10000 | Yes |
| H007 | Female | 65+ | Secondary | Widowed | No | Living Alone | Rental | Public | Retired | <$10000 | No |
| H008 | Female | 65+ | Primary or Below | Widowed | No | Living Alone | Rental | Sub-divided Unit | Part-time | <$10000 | No |
| H009 | Female | 65+ | Primary or Below | Married | Buddhism | Living with Family | Rental | Public | Retired | <$10000 | Yes |
| H010 | Female | 15-24 | Secondary | Single | No | Living with Family | Private | Public | Full-time | $10001-20000 | Yes |
| H011 | Female | 25-44 | Secondary | Single | No | Living with Family | Rental | Private | Full-time | $10001-20000 | Yes |
| H012 | Female | 45-64 | Secondary | Married | Buddhism | Living with Family | Rental | Private | Full-time | >$50000 | No |
| H013 | Male | 25-44 | Tertiary | Single | No | Living with Family | Private | Private | Full-time | $30001-40000 | No |
| H014 | Male | 15-24 | Tertiary | Single | No | Living with Family | Private | Private | Full-time | $20001-30000 | No |
| H015 | Female | 45-64 | Secondary | Married | No | Living with Family | Rental | Public | Part-time | <$10000 | Yes |
| H016 | Female | 15-24 | Tertiary | Single | No | Living with Family | Private | Private | Full-time | $10001-20000 | No |
| H017 | Male | 25-44 | Tertiary | Single | MP | Living with Family | Private | Public | Full-time | $30001-40000 | No |
| H018 | Female | 25-44 | Tertiary | Single | Christian | Living with Family | Private | Private | Full-time | $20001-30000 | No |
| H019 | Female | 25-44 | Secondary | Single | No | Living with Family | Private | Private | Full-time | $10001-20000 | No |
| H020 | Male | 45-64 | Tertiary | Married | Christian | Living with Family | Private | Private | Full-time | >$50000 | Yes |
| H021 | Female | 45-64 | Tertiary | Divorced | Christian | Living with Family | Rental | Private | Full-time | $20001-30000 | No |
| H022 | Female | 45-64 | Secondary | Divorced | Christian | Living with Family | Rental | Public | Part-time | $10001-20000 | Yes |
| H023 | Male | 25-44 | Secondary | Single | No | Living with Family | Private | Private | Full-time | <$10000 | Yes |
| H024 | Male | 45-64 | Primary or Below | Widowed | Christian | Living with Family | Private | Private | Full-time | $20001-30000 | No |
| H025 | Male | 45-64 | Secondary | Married | No | Living with Family | Private | Private | Full-time | $20001-30000 | Yes |
| H026 | Female | 45-64 | Primary or Below | Married | Buddhism | Living with Family | Rental | Public | Full-time | $10001-20000 | Yes |
| H027 | Male | 65+ | Primary or Below | Married | No | Living with Family | Rental | Sub-divided Unit | Full-time | $10001-20000 | No |
| H028 | Male | 65+ | Primary or Below | Married | No | Living with Family | Rental | Private | Unemployed | <$10000 | No |
| H029 | Male | 45-64 | Primary or Below | Married | No | Living with Family | Rental | Private | Part-time | $10001-20000 | No |
| H030 | Male | 25-44 | Secondary | Single | No | Living with Family | Private | Subsidised | Full-time | $20001-30000 | No |

**Appendix 3: English Translation of the Interview Guide**

**Jockey Club School of Public Health and Primary Care**

**Faculty of Medicine**

**The Chinese University of Hong Kong**

An Exploratory Study on the
Potential Bolt-on Items onto the EQ-5D Adult Set in Hong Kong Context

**Qualitative Individual Interview: Interview Guide**

**Sampling Criteria**

- Aged 18 or above
- Able to Communicate in Cantonese
- Hong Kong Permanent Residents
- Cognitively Healthy: Being Able to Understand and Sign Consent Form

1. **The Perception on ‘Health’ and ‘Health-related Quality of Life’(10-15 Minutes)**

- In your opinion, what is ‘health？
  - What characteristics may a healthy/unhealthy person possess?
  - World Health Organisation(WHO)’s Definition of Health:
    *‘a state of complete physical, mental and social well-being and not merely the absence of disease or infirmity’*
- In your opinion, how does health correlate/ affect daily life?
  - How does health impact on Health-related Quality of Life or any aspects in daily life?
  - Any factor(s) that will impact the relationship or linkage between health and daily life？

1. **5-level EuroQol-5D（EQ-5D-5L): Feedback and Comments(15-20 Minutes)**

- In your opinion, can the EQ-5D-5L instrument reflect your health status？
  - Does the EQ-5D-5L report important indicators of Health-related Qualify of Life？
  - Is there too many/too few/ overlapping with the 5-dimension design? Is there any need to include/ exclude any certain dimension(s)?
  - If you do not perceive yourself as experiencing ‘full health’, do you think the EQ-5D-5L can explain the reason behind? Can the gap be revealed easily?
- In your opinion, do you think the Visual Analog Scale(VAS) is an accurate health utility measurement？
  - If you did not report the full score 100, does the score deducted related to the five dimensions included in the EQ-5D-5L?
  - Besides the five dimensions, did you consider other factors in your VAS response？

1. **5-level EuroQol-5D（EQ-5D-5L）: Potential Bolt-on Dimensions(15-20Minutes)**

- In your opinion, which of the following item(s) may improve the current EQ-5D-5L instrument？How do you interpret this/ these dimension(s)?

| EQ-5D Exploratory Bolt-on |
| --- |
| - Satisfaction |
| - Speech/ Cognition |
| - Interpersonal Relationships |
| - Hearing |
| - Vision |
| - Energy |
| - Sleep |
| - Appetite |

| WHOQOL-BREF (Hong Kong) | |
| --- | --- |
| Physical Health | - How much do you need any medical treatment to function in your daily life? |
|  | - How satisfied are you with your capacity for work |
| Psychological | - Acceptance for Bodily Appearance |
|  | - How often do you have negative feelings such as blue mood, despair, anxiety, or depression? |
|  | - How much do you enjoy life? |
|  | - How satisfied are you with yourself? |
|  | - To what extent do you feel your life to be meaningful? |
|  | - How well are you able to concentrate? |
|  | - To what extent do you feel being respected in daily life? |
| Social Relationships | - How satisfied are you with your personal relationships? |
|  | - How satisfied are you with the support you get from your friends? |
|  | - How satisfied are you with your sex life? |

- Considering people around you, (Different age: young adult, middle-aged or elderly; Different life stage: studying, building a family, employed, retired; Different socio-background/education/occupation; etc.), do you think they have other feedback/ opinion on this list of bolt-on items?

1. **Do you have any comments or opinions on the EuroQol-5D instrument or the potential bolt-on items？**
